# Crater lake cichlids individually specialize along the benthic–limnetic axis

**DOI:** 10.1002/ece3.1015

**Published:** 2014-03-07

**Authors:** Henrik Kusche, Hans Recknagel, Kathryn Rebecca Elmer, Axel Meyer

**Affiliations:** 1 Department of Biology, Chair of Zoology and Evolutionary Biology, University of KonstanzKonstanz, 78457, Germany; 2 International Max Planck Research School for Organismal Biology, University of KonstanzKonstanz, 78457, Germany

**Keywords:** Benthic–limnetic axis, divergent selection, ecological opportunity, individual specialization, parallel evolution, phenotype-diet correlation

## Abstract

A common pattern of adaptive diversification in freshwater fishes is the repeated evolution of elongated open water (limnetic) species and high-bodied shore (benthic) species from generalist ancestors. Studies on phenotype-diet correlations have suggested that population-wide individual specialization occurs at an early evolutionary and ecological stage of divergence and niche partitioning. This variable restricted niche use across individuals can provide the raw material for earliest stages of sympatric divergence. We investigated variation in morphology and diet as well as their correlations along the benthic-limnetic axis in an extremely young Midas cichlid species, *Amphilophus tolteca,* endemic to the Nicaraguan crater lake Asososca Managua. We found that *A. tolteca* varied continuously in ecologically relevant traits such as body shape and lower pharyngeal jaw morphology. The correlation of these phenotypes with niche suggested that individuals are specialized along the benthic-limnetic axis. No genetic differentiation within the crater lake was detected based on genotypes from 13 microsatellite loci. Overall, we found that individual specialization in this young crater lake species encompasses the limnetic-as well as the benthic macro-habitat. Yet there is no evidence for any diversification within the species, making this a candidate system for studying what might be the early stages preceding sympatric divergence.

A common pattern of adaptive diversification in freshwater fishes is the repeated evolution of open water (limnetic) species and of shore (benthic) species. Individual specialization can reflect earliest stages of evolutionary and ecological divergence. We here demonstrate individual specialization along the benthic–limnetic axis in a young adaptive radiation of crater lake cichlid fishes.

## Introduction

Understanding how ecological, morphological, and genetic variation is created and maintained is of central interest in evolutionary biology. During the process of incipient ecological speciation, disruptive selection can be reflected by individual ecological specialization stemming from intraspecific competition for resources (Schluter 2000; Bolnick and Fitzpatrick 2007; Nosil 2012). Individual specialization indicates restricted individual niche exploration relative to the population overall (Bolnick et al. 2003), most probably due to trade-offs that constrain an individual's resource use (e.g., Hatfield and Schluter 1999). This should therefore translate into significant phenotype-environment correlations at the individual level (Schluter 2000; Martin and Pfennig 2009). Individual specialization has important eco-evolutionary consequences because the variation in interindividual niche use directly affects the degree of intraspecific competition and therefore the capacity for diversification and speciation (Bolnick et al. 2003). It has been proposed that through individual specialization, frequency-dependent processes are facilitated that potentially lead to the broadening of the resource spectrum (Van Valen 1965; Svanbäck and Bolnick 2007), the evolution and maintenance of polymorphisms (Smith and Skúlason 1996; Swanson et al. 2003) and finally ecological speciation in sympatry (Dieckmann and Doebeli 1999; Bolnick and Fitzpatrick 2007; Bolnick 2011). Therefore, putative early cases of divergence that are studied at the individual level are the most relevant context for analyzing incipient events of speciation and adaptive radiation (Schluter 2000; Bolnick et al. 2003; Matthews et al. 2010; Bolnick 2011).

**Figure 1:**
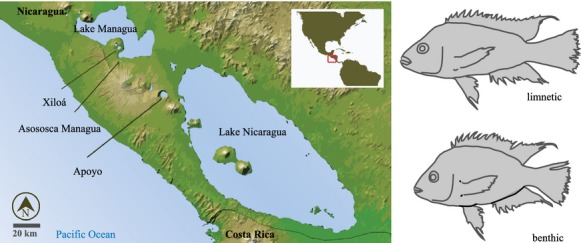
Divergence along the benthic–limnetic axis in Nicaraguan crater lakes. In western Nicaragua (Central America), several crater lakes have been colonized independently by Midas cichlids from the great lakes (Lake Managua and Lake Nicaragua). Midas cichlids in crater lakes Apoyo and Xiloá have speciated along the benthic-limnetic axis. The schematic drawings indicate high-bodied “benthic” specimens that rather live and forage in the littoral zone, while the slender-bodied “limnetic” individuals explore the open water column. This study focuses on *A. tolteca* from the small and young crater lake Asososca Managua.

Instances of parallel evolution, where similar phenotypes arise independently in different environments from a recent common ancestor, provide strong evidence for natural selection in driving diversification (Schluter and Nagel 1995; Elmer and Meyer 2011). In freshwater fishes, one major avenue of parallel diversification often occurs along the benthic–limnetic axis (Fig. 1), with benthic ecomorphs being characteristically high-bodied, and limnetics being of rather fusiform (elongated) body shape, and these alternative body forms are associated with a benthic (shore-associated) versus a limnetic (open and deep water) life style (Webb 1982, 1984; Robinson and Wilson 1994; Robinson and Schluter 2000). The best-studied examples of ecomorphological differentiation along the benthic–limnetic axis are fishes in postglacial lakes, such as the three-spine stickleback, *Gasterosteus aculeatus* (Schluter and McPhail 1992; McPhail 1994), whitefish, *Coregonus spec*. (Hudson et al. 2005; Østbye et al. 2006), arctic char, *Salvelinus alpinus* (Malmquist et al. 1992; Jonsson and Jonsson 2001), and perch, *Perca fluviatilis* (Svanbäck and Eklöv 2003) that have diversified, often in multiple independent instances, into benthic and limnetic forms.

Neotropical Midas cichlids (*Amphilophus* cf. *citrinellus* Günther) from the Nicaraguan crater lakes also mirror this pattern of benthic–limnetic diversification, even in sympatry, which makes this a model system for studying adaptive radiation and parallel evolution (Meyer 1990a,b1990b; Barluenga et al. 2006; Elmer et al. 2010a; Franchini et al. in press). In western Nicaragua, several crater lakes formed by accumulation of rain and ground water in isolated volcanic calderas. In rare, and likely independent events, a putative generalist Midas cichlid ancestor from the Nicaraguan great lakes colonized these newly formed crater lakes and exploited niches uniquely available there (Barluenga et al. 2006; Elmer et al. 2010a, 2013). In some crater lakes, Midas cichlids have speciated sympatrically along the benthic–limnetic axis (e.g., in lakes Apoyo and Xiloá endemic limnetic species *Amphilophus zaliosus* (Barlow and Munsey 1976) and *Amphilophus sagittae* (Stauffer and McKaye 2002), respectively), but how this differentiation proceeded ecologically still remains largely unexplored.

Quantifying the individual specialization that may eventually promote speciation through disruptive selection requires examining individuals within variable populations that have not yet speciated (Smith and Skúlason 1996; Bolnick et al. 2003; Swanson et al. 2003; Martin and Pfennig 2009; Bolnick 2011). We assessed ecological variation, individual specialization, and divergence along the benthic–limnetic axis in *Amphilophus tolteca* (Recknagel et al. 2013), an extremely young species of Midas cichlids endemic to the maximally 1245 year old crater lake Asososca Managua (Pardo et al. 2008). It has been suggested, though not previously tested, that this variable species has differentiated into macrohabitats, and that discrete morphs (or even species) might have evolved within such a short period of time (Elmer et al. 2010a). Here, we comprehensively tested this hypothesis by assessing genetic clustering and trait distributions of ecomorphology and diet that are known to differ along the benthic–limnetic axis: Body depth (e.g., Webb 1982, 1984), body and lower pharyngeal jaw (LPJ) size and shape (e.g., Muschick et al. 2012), and stable isotope signatures of nitrogen (*δ*^15^N) and carbon (*δ*^13^C) (e.g., Post 2002). We expected that *A. tolteca* individuals with elongated morphologies and more delicate pharyngeal jaws would rely more on a limnetic diet (i.e., are depleted in *δ*^13^C) compared with high-bodied individuals. Additionally, body shape and body height index (BHI) of *A. tolteca* was compared with the benthic–limnetic divergent Midas cichlid communities of crater lakes Apoyo and Xiloá. Finally, we demonstrated that this young Midas cichlid species has not diverged into discrete ecomorphs or subpopulations along the benthic–limnetic axis as has been previously suspected (Elmer et al. 2010a) and rather represents continuous ecological variation and individual specialization.

## Materials and Methods

### Specimen collection

*Amphilophus tolteca* specimens (*n *=* *190) were collected by gill-netting in 2010 and 2012 from various locations at the northeast shore of Asososca Managua (12°08.390′ N 086°18.792′ W). In the field, standardized photographs were taken from directly above using a tripod and a Canon Power Shot D10 digital camera (Canon, Tokyo, Japan). All specimens were taken as vouchers (head or whole body) and stored in 70% ethanol. Fin and muscle tissue samples for DNA analysis were preserved in pure ethanol.

### Body shape and body height index analyses

Body shape variation within *A. tolteca* was analyzed using geometric morphometric approaches. Eighteen landmarks (LM) describing body shape were digitized from photographs (*n *=* *190) in tpsDig v. 2.16 (Rohlf 2010a) by a single investigator (Fig. S1). Allometry-corrected body shape analyses were performed in MorphoJ1.05a (Klingenberg 2011) following Elmer et al. (2010a). Variation in individual body shapes was investigated using principal component analysis (PCA). The thin plate spline technique (Dryden and Mardia 1998) was used to visualize body shape changes associated with principal components (PCs). To quantitatively assign each specimen along the benthic–limnetic axis in terms of body shape, we defined the body height index (BHI). BHI is the relative fraction of body height as a function of standard length. BHI was calculated from interlandmark distances that were obtained in PAST v. 2.16 (Hammer et al. 2001) for each individual. The interlandmark distance between LM 6 and LM 9 (Fig. S1) was used as a proxy for body height. Using major axis regression, no allometric effects of BHI were detected for the focal species [95% confidence interval ranged between −0.002 and 0.063; package smatr (Warton et al. 2012)]. Therefore, uncorrected BHI was used in downstream analyses.

### Lower pharyngeal jaw size and shape analyses

Lower pharyngeal jaws (LPJ) were extracted from the head of 96 ethanol-preserved *A. tolteca* specimens. External characteristics of LPJs (lateral horn width, jaw length, keel depth; Fig. S1) were measured using a digital calliper, and jaws were weighted to the nearest mg using a precision scale. In a complementary approach, LPJs were placed in an agarose gel chamber and the dentition side was photographed from directly above using a tripod and a Canon Power Shot D10 digital camera. Twenty-four homologous landmarks, consisting of 12 fixed and 12 semilandmarks, were defined that describe external LPJ shape as well as the shape of the dentigerous area. Digitization was carried out in tpsDig v. 2.16 (Rohlf 2010a) by a single investigator from the photograph of each specimen (Fig. S1). Semilandmarks were slid in tpsRelw v. 1.49 (Rohlf 2010b) in orthogonal projection mode with 10 iterations. Slid semilandmarks were treated as true homologous landmarks in MorphoJ1.05a (Klingenberg 2011). Object symmetry was taken into account, and the symmetric component of shape variation only was considered as our trait of interest (Klingenberg et al. 2002). Allometric effects on LPJ shape were corrected by regressing Procrustes coordinates on centroid size (12.47% explained; *P *<* *0.0001). Regression residuals were used in downstream analyses that were conducted analogous to body shape analyses.

### Long-term analysis of diet: inferences from stable isotopes

Stable isotope measurements from fish muscle tissue provide a long-term record of feeding history in aquatic ecosystems and are therefore ideal to study interindividual variation in feeding history. In the lacustrine environment, stable carbon (*δ*^13^C) signatures measured in consumers inform about the carbon source of the prey items, with benthic origin being usually enriched in *δ*^13^C compared with limnetic origin (France 1995; Hecky and Hesslein 1995; Zanden and Rasmussen 1999; Zanden et al. 1999; Post 2002). Stable nitrogen (*δ*^15^N) indicates the relative trophic level (France 1995; Hecky and Hesslein 1995; Zanden and Rasmussen 1999; Zanden et al. 1999; Post 2002; Bolnick 2011). A small piece of muscle tissue was extracted from dorsal musculature of 73 ethanol-preserved specimen of *A. tolteca* and dried for ca. 48 h at 55°C. Samples were ground in individual sealed tubes, and a 1–1.5 mg subsample was used for isotope analyses. Gas chromatography combustion isotope ratio mass spectrometry (GC-C-IRMS) was performed at the Limnological Institute (University of Konstanz). *δ*^13^C values were corrected for lipid content following Kiljunen et al. (2006).

### Assessment of individual ecological specialization in Asososca Managua

To assess the correlations of diet and eco-morphological variables at the individual level, linear regression analyses were conducted in R (R Core Team 2012). Stable isotope signatures were tested for correlation with LPJ weight (*n *=* *54) and BHI (*n *=* *73) separately. To test for a correlation of jaw-and overall body morphology, BHI and LPJ weights (*n *=* *95) were investigated. LPJ weight was selected because it highly correlates with other LPJ variables (see Fig. S2) and therefore is a proxy of jaw hypertrophy, also see Muschick et al. (2011). BHI was used because it is highly correlated with PCs 1–3 of the body shape analysis and summarizes body elongation (and likely reflects individual specialization along the benthic–limnetic axis, see also Fig. S2).

If disruptive selection were driving separation along the benthic–limnetic axis, then signatures of selection such as bimodality should be identifiable in trait distributions (Schluter 2000; Rundle and Nosil 2005). To test whether the trait distributions were best explained by one or two components and whether there was discontinuous variation, mixture analysis was conducted for all traits separately. The mixture analysis comprised the dip test for unimodality (Hartigan and Hartigan 1985), the Anscombe–Glynn test for platykurtosis (Anscombe and Glynn 1983) and an expectation–maximization (EM) algorithm-based approach (McLachlan and Peel 2000) implemented in the mixtools package (Benaglia et al. 2009) that evaluated whether one or two components were most likely. Normal probability plots and Shapiro–Wilk tests (Shapiro and Wilk 1965) were also consulted to infer possible deviations from a single normal distribution.

To consider all variables simultaneously, a model-based clustering approach was applied to a subset of 54 individuals for which the following seven measurements were available: BHI, allometry-corrected LPJ weight, depth, width, and length, and stable isotope signatures *δ*^13^C and *δ*^15^N. This Bayesian approach implemented in the mclust package (Fraley et al. 2012) was used to identify the optimal number and cluster type for this data set from a range of parameterized Gaussian mixture models for 1–9 clusters and varying covariance matrices.

### Assessment of neutral genetic differentiation

Thirteen microsatellite loci were amplified and genotyped for 118 *A. tolteca* individuals (M1M, M2, M7, M12 (Noack et al. 2000), UNH002 (Kellogg et al. 1995), UNH011, UNH012, UNH013 (McKaye et al. 2002), Abur45, Abur82, Abur151 (Sanetra et al. 2009), Burtkit F 474/R672 (Salzburger et al. 2007), TmoM7 (Zardoya et al. 1996) following previously published methods. Descriptive statistics, inbreeding coefficient F_IS_, and gene diversity were calculated in FSTAT v. 2.9.3.2 (Goudet 1995). Rarefied allelic richness was assessed in HP–Rare v. June-6-2006 (Kalinowski 2005). Structure v. 2.3.3 (Pritchard et al. 2000) was run for 500,000 generations after 50,000 generations burnin. Five independent runs were assessed for each *k* = 1–5 to determine any intraspecific genetic structuring.

### Across-lake comparison of body shape and Body Height Index

Principal component analysis (PCA) was performed to compare body shape across different Midas cichlid species diverged along the benthic–limnetic axis (Elmer et al. 2010a). For this approach, additional data derived from previous expeditions were consulted for lakes Apoyo (six species; 488 specimens; body shape and BHI), Xiloá (four species; 460 specimens; body shape and BHI), and Asososca Managua (96 specimens; BHI). In the combined sample of 1,138 individuals, body shape showed significant allometric effects (6.12% of shape was explained by centroid size; *P *<* *0.0001); thus, the size-corrected shape data (regression residuals) were used in downstream analyses. Two species, *A. zaliosus* from Lake Apoyo (Barlow and Munsey 1976) and *A. sagittae* from Lake Xiloá (Stauffer and McKaye 2002), are limnetic species (Elmer et al. 2010a), whereas the other Midas cichlid species are benthics.

## Results

### Ecological variation along the benthic-limnetic axis

#### Body shape

**Figure 2:**
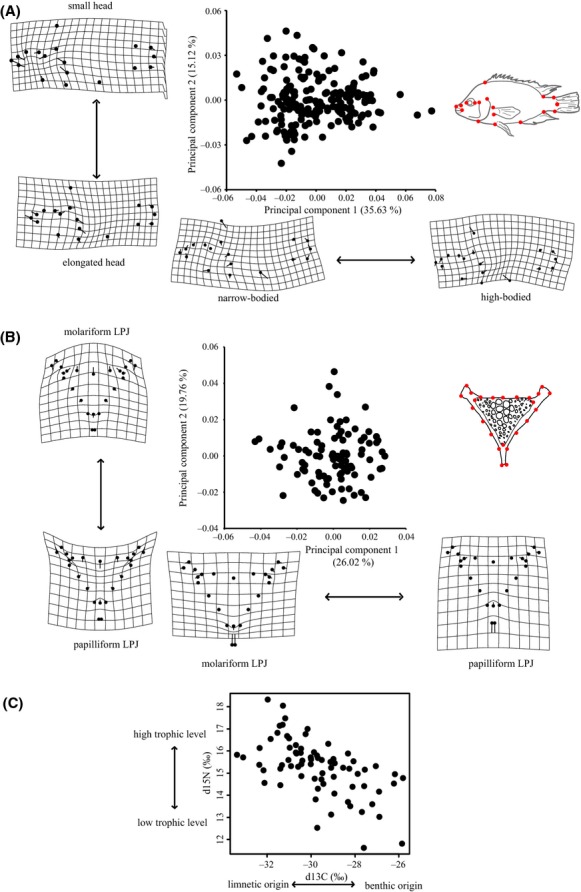
Midas cichlid variation in Asososca Managua along the benthic-limnetic axis. (A) Body shape: Biplots of PCs 1 and 2 that encode for 50.75% of the total body shape variation. The line terminus in the transformation grids depicts the shape changes from the overall mean associated with each PC (scale factor of 0.1 in positive and negative directions). Positive values on PCs 1 and 2 indicate rather high-bodied (benthic-like) individuals, whereas negative values on both PCs overall indicate rather elongated (limnetic-like) individuals. See Fig. S1 for detailed landmark definitions. (B) lower pharyngeal jaw (LPJ) shape. Biplots of PCs 1 and 2 that encode for 45.78% of the total LPJ shape variation. Deformation grids depict a scale factor of 0.1 in positive and negative directions on both PCs. Positive values on PC1 and negative values on PC2 indicate rather delicate LPJ-morphology (“papilliform”) individuals. See Fig. S1 for landmark definitions. (C) Biplots of stable isotopes *δ*^15^N and *δ*^13^C. The more enriched an individual is in *δ*^15^N, the higher is its trophic level. Individuals that forage in the benthic habitat are usually enriched in *δ*^13^C and *δ*^13^C is usually depleted in limnetic ecotypes.

Body morphology of *A. tolteca* ranged from typically benthic-shaped high-bodied individuals with relatively small heads to typically limnetic-shaped elongated individuals (Fig. 2A). Along the first two principal components, which together describe 50.75% of the shape variation, benthic-like fish are mainly characterized by a dorsoventral expansion and anterior-posterior contraction of the transformation grid (LM 6 and LM 9–12) and a relative shortening of the caudal peduncle (LM 13–15; Fig. 2A). Accordingly, limnetic-like fish are mainly characterized by a dorsoventral compression of the grid, a relative elongation of the caudal peduncle and the head (LM 1–5, 7–8, 17–18), compared with benthic-like fish.

#### Lower pharyngeal jaw shape

A range of LPJ-morphologies were detected within *A. tolteca,* which varied along the benthic–limnetic axis as typified by the extent of characteristic robust molariform or slender papilliform shape (Fig. 2B) (e.g., Meyer 1990a,1990b). PC1 and PC2 captured 45.78% of the total variation in LPJ shape, respectively. Positive PC1 scores reflected jaws that had long and more delicate horns. PC2 was a good indicator of the overall LPJ morphology, with positive values representing LPJ margins warped toward the outside (LM 4–8, 12–14 and LM 19–21) as well as wider and sturdier lateral horns (LM 1–4, LM 21, and LM 8–12), and a relatively enlarged dentition area (as defined by LM 3–8 and LM 22–24; Fig. 2B), that is, more molariform.

#### Long-term analysis of diet: inferences from stable isotopes

The long-term analysis of diet revealed considerable variation in stable isotope signatures within the focal species (Fig. 2C). The *δ*^15^N signatures ranged from 11.62‰ to 18.33‰ (mean = 15.25‰; SD = 1.29 ‰; Δ 6.71 ‰) and the *δ*^13^C signatures ranged from −33.37‰ to −25.82 ‰ (mean = −29.69‰; SD = 1.75 ‰; Δ 7.55‰), indicating interindividual variation of consistent ecological resource use.

### Relationships between diet and eco-morphological traits

**Figure 3:**
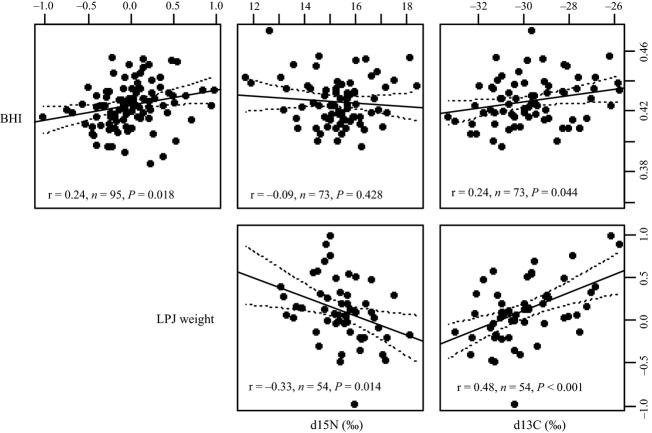
Correlation of eco-morphological variables and diet. Pair-wise correlations of the eco-morphological variables body height index (BHI), lower pharyngeal jaw (LPJ) weight, *δ*^15^N, and *δ*^13^C are depicted together with the outcome of linear regression analysis. The solid line indicates the regression line. The 95% confidence interval is indicated by the dotted lines. BHI was positively correlated with LPJ weight and *δ*^13^C. LPJ weight was negatively correlated with *δ*^15^N and positively correlated with *δ*^13^C. Limnetic-like individuals (indicated by low BHI and LPJ weight values) tend to feed on a slightly higher trophic level (enriched in *δ*^15^N) than benthic-like individuals (high BHI values). Further, elongated fish with delicate LPJ morphologies are less enriched in *δ*^13^C, likely indicative of their limnetic life style.

Individual specialization can be inferred when morphological adaptations are correlated with ecology (Matthews et al. 2010). We found that the relevant variation in body morphology along the benthic–limnetic axis (as indicated by body height index [BHI]) largely corresponded to overall jaw morphology (LPJ weight), which itself reflected feeding ecology as measured by stable isotope signatures (Fig. 3). Specifically, BHI and LPJ weight were positively correlated (linear model: *r* = 0.24, *n *=* *95, *P *=* *0.018). LPJ weight was negatively correlated with trophic level, *δ*^15^N, (*r* = −0.33, *n *=* *54, *P *=* *0.014) and was strongly positively correlated with *δ*^13^C, which typically characterizes preferential use of benthic–limnetic macrohabitats (Post 2002) (*r* = 0.48, *n *=* *54, *P *<* *0.001). BHI generally showed the same trend as LPJ weight in being correlated with niche inferred from stable isotopes, although the effect was overall less significant (*δ*^15^N: *r* = −0.09, *n *=* *73, *P *=* *0.428; *δ*^13^C (*r* = 0.24, *n *=* *73, *P *=* *0.044; Fig. 3).

### Divergence within *A. tolteca*

We tested whether the distributions of ecomorphological traits in *A. tolteca* were best described by multiple components and distinct clusters. Such bimodality or clustering suggests sympatric ecological diversification, with multiple fitness peaks promoting phenotypic divergence (Schluter 2000; Rueffler et al. 2006; Hendry et al. 2009b; Elmer et al. 2010b). Using mixture analyses, we found that the distributions of all LPJ variables, stable isotopes, and BHI were each best explained by a single component (i.e., unimodality cannot be rejected for bimodality; Table 1). When all traits were combined (BHI, LPJ weight, length, depth, and width, stable isotopes; *n *=* *54 individuals with complete dataset) three spherical, clusters with varying volume were slightly higher supported (Δ BIC was −10 and −12 to the next best models with two and one cluster respectively). However, principal component analysis on the same set of individuals did not confirm any such clustering (Fig. S2).

**Table 1:** Distributions of ecologically relevant traits within *Amphilophus tolteca*. Mixture analyses were performed to determine the most likely number of components in each variable. All traits were supported by continuous distributions

Character	Sample size	Dip test		Anscombe-Glynn	Shapiro-Wilk	Mixture analysis	
	*n*	Dip	*P*	*P*	*P*	*n* components	*P*
LPJ weight	96	0.025	>0.95	0.26	0.66	1	0.68
LPJ width	96	0.028	>0.5	0.07	0.14	1	0.43
LPJ length	96	0.033	>0.5	0.6	0.25	1	0.74
LPJ depth	96	0.027	>0.9	0.68	0.85	1	0.97
*δ*^15^N	73	0.024	>0.99	0.12	0.16	1	0.23
*δ*^13^C	73	0.029	>0.5	0.44	0.41	1	0.61
BHI	286	0.015	>0.5	0.31	0.15	1	0.33

BHI, body height index; LPJ, lower pharyngeal jaw.

A relatively low level of genetic polymorphism was identified in the multilocus analysis of 13 polymorphic microsatellites. The number of alleles per locus ranged between 2 (Abur 151) to 14 (M7 & UNH013; Table S1). The inbreeding coefficient (F_IS_) was 0.051. Gene diversity was 0.549, and allelic richness was 7.28. There was no evidence for genetic clustering within *A. tolteca* (*k* = 1: ln = −3347.9 ± 0.07; *k* = 2: ln = −3331.3 ± 0.43; *k* = 3: ln = −3327.2 ± 1.68; *k* = 4: ln = −3334.1 ± 0.85, *k* = 5: ln = −3334.4 ± 2.76). Individuals could not be assigned into different genetic clusters when a priori fixing the number of population to two (*K* = 2), as indicated by individual membership values close to 0.5 (*Q* = 0.4–0.6). This suggested a single panmictic population.

### Across-lake comparison of body shape and Body Height Index

**Figure 4:**
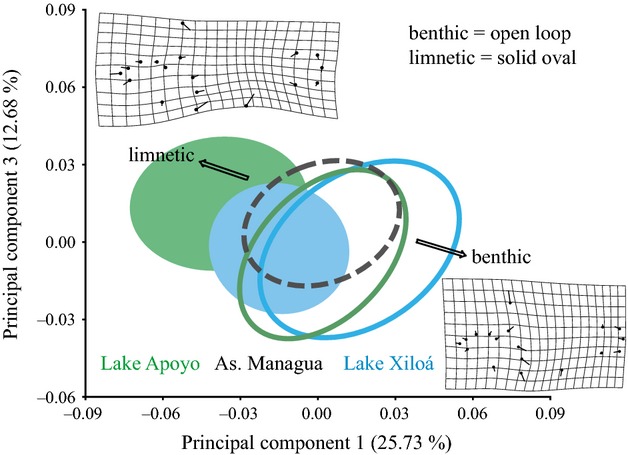
Midas cichlid body shape differentiation along the benthic-limnetic axis. The divergence in body shape between benthic and limnetic species from crater lakes Apoyo (six species) and Xiloá (four species) is demonstrated and contrasted to the focal species from Asososca Managua. Depicted are 90% confidence ellipses along PCs 1 and 3 from a joint principal component analysis of body shape. Shape changes along PCs 1 and 3 (scale factor = 0.1) are indicated by thin plate splines. The line terminus refers to the shape change along a particular principal component, compared with the average shape (black dot). The shape change of these axes corresponds to typical body shape differences along the benthic-limnetic axis (relative body height and elongation as well as snout bluntness). Positive values on PC1 and negative values on PC3 indicate rather benthic individuals. *Amphilophus tolteca* (90% confidence ellipse indicated by broken line) overlaps with benthic and limnetic species from Lakes Apoyo and Xiloá, and in contrast to Lakes Apoyo and Xiloá, no intralacustrine divergence is observed.

We contrasted the variation in *A. tolteca* body shape with the ecomorphological differentiation found in the older crater Lakes Apoyo (up to 24,000 years old) and Xiloá, (about 6000 years old) that both house multiple species along the benthic–limnetic axis (Elmer et al. 2010a). The PCA of all available specimens from Asososca Managua, Apoyo and Xiloá revealed divergence in body shape of Lakes Apoyo (five benthic, one limnetic species), and Xiloá (three benthic, one limnetic species; Fig. 4), but not in *A. tolteca*. PC1 and PC3 together accounted for 38.41% of the total variation and represented a typical change of body shape along the benthic–limnetic axis, particularly with respect to body elongation, body height, and relatively more posterior dorsal and anal fin placement (Fig. 4). The focal species overlaps with benthic and limnetic species from both other lakes.

**Figure 5:**
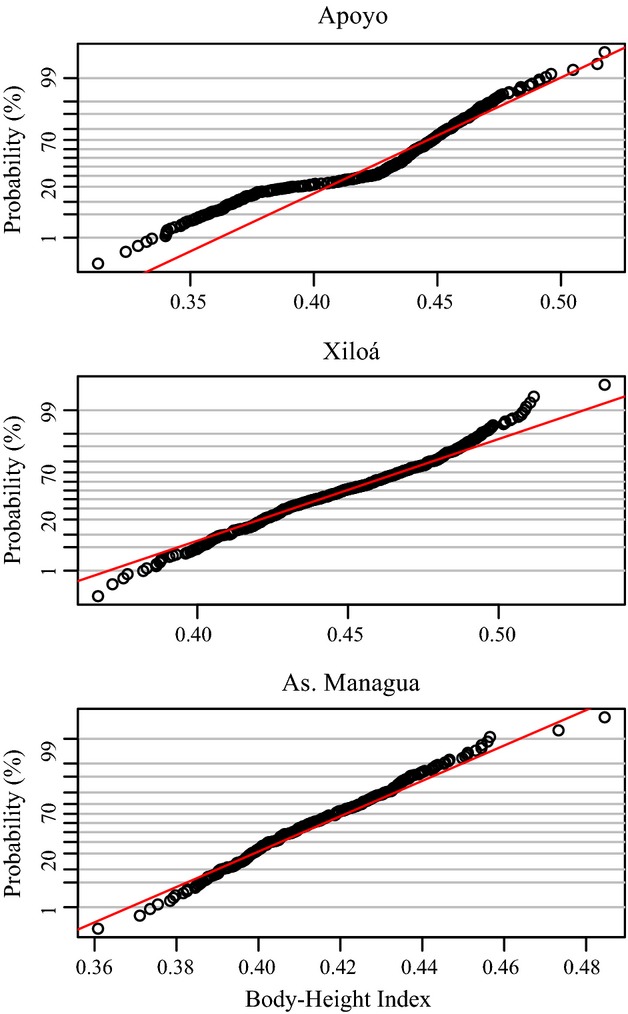
Distribution of body height index across lakes. Normal probability plots with cumulative proportions of observed versus expected proportions of body height index (BHI) in comparison to a single normal distribution (red line) for lakes Apoyo, Xiloá and Asososca Managua. Trait distributions for lakes Apoyo and Xiloá are best explained by two components while in Asososca Managua they are most likely explained by one (see section “Results”).

The body height index (BHI) is a summary statistic of body shape where lower values mean greater elongation and therefore shallower bodies. BHI conformed to a bimodal distribution in lakes Apoyo and Xiloá that both house multiple species, but not in Asososca Managua (Apoyo: *n *=* *488, *P *<* *0.001; Xiloá: *n *=* *460, *P *<* *0.004; Asososca Managua: *n *=* *286, *P *=* *0.33; see also Fig. 5 for visualization).

## Discussion

In this study, we focussed on the Midas cichlid species, *A. tolteca,* endemic to Nicaragua's youngest crater lake, Asososca Managua. We detected significant phenotype-diet correlations along the benthic–limnetic axis, likely indicating individual specialization in habitat use. We did not detect bimodality in ecologically relevant traits and no genetic divergence in sympatry. This is in contrast to crater lake Apoyeque, in which Midas cichlid ecomorphs have rapidly diverged in sympatry in a crater lake ca. 1800 years old (Elmer et al. 2010b; Manousaki et al. 2013), or the older crater lakes Apoyo or Xiloá with multiple endemic species (Elmer et al. 2010a).

### Variability and individual specialization along the benthic-limnetic axis

Our study documented interindividual variation in body shape and LPJ-related size and shape, as well as in stable isotope signatures indicative of niche. A variety of body shape phenotypes are present in *A. tolteca*, ranging from elongated to high-bodied individuals (Fig. 2A). These body shapes are typically indicative of divergence along the benthic–limnetic axis in Midas cichlids [Figs. 5 and Elmer et al. (2010a); see also “arrow shaft metaphor” that describes the body shape of limnetic cichlids in Fryer and Iles (1972)]. Similarly, the variation in LPJ morphology ranges from delicate (papilliform) to rather robust and sturdy (molariform) LPJs (Fig. 2B). The stable isotope signatures (Fig. 2C) in this species spanned multiple trophic levels (*δ*^15^N), in agreement with stable isotope foraging theory (Zanden and Rasmussen 1999; Zanden et al. 1999; Post 2002), and suggested the long-term exploration of different macro-habitats (*δ*^13^C) within this lake (France 1995; Hecky and Hesslein 1995; Zanden and Rasmussen 1999; Zanden et al. 1999; Post 2002). It is generally found that enrichments of ∼3 ‰ in *δ*^15^N correspond to the shift of a single trophic level, that is these differences would be expected in a consumer prey relationship, whereas enrichments of *δ*^13^C would be less important in this regard (0–1 ‰ enrichment along a single trophic level) (Zanden and Rasmussen 1999; Zanden et al. 1999; Post 2002). However, even within a single body of water, *δ*^13^C indicates the primary producers upon which a consumer feeds, since benthic algae are typically enriched in *δ*^13^C relative to free floating phytoplankton of the open water column (France 1995; Hecky and Hesslein 1995; Zanden and Rasmussen 1999; Zanden et al. 1999; Post 2002). Our data suggest that *A. tolteca* covers at least two trophic levels and reveals considerable variation in the carbon source of its prey items along the benthic–limnetic axis. However, without incorporating the isotopic composition of the prey items, individual specialization cannot be unambiguously concluded from the consumer's *δ*^13^C alone because variation in the prey carbon source can cause a similar pattern in the consumer (Matthews and Mazumder 2004).

More direct evidence for individual specialization stems from our demonstration of ecological relevance of individual morphologies (Fig. 3). We showed that the correlation of morphological and ecological features of individuals conforms to what is typically associated with restricted habitat use along the benthic–limnetic axis (Webb 1982, 1984; Robinson and Wilson 1994; France 1995; Hecky and Hesslein 1995; Taylor 1999; Zanden and Rasmussen 1999; Zanden et al. 1999; Mousseau et al. 2000; Post 2002), suggesting individual specialization to contrasting macro-habitats. Consistently, the typically benthic fish were higher-bodied and less arrow-like-shaped, had more robust pharyngeal jaws, fed at a lower trophic level, and more frequently exhibited a littoral carbon source compared to limnetic fish (Fig. 3, Fig. S2). In other words, the difference in diet is significantly associated with ecomorphological traits, most importantly with LPJ weight and overall body elongation (BHI). LPJ weight, as a proxy for LPJ hypertrophy, was correlated with long-term diet in terms of stable isotope signatures (Fig. 3). This means that the heavier a LPJ of a given individual is, the less enriched this individual will be in *δ*^15^N and the more likely its carbon source will be of a benthic origin. This is an important consideration since the robustness of the LPJ limits the food sources a fish can explore, that is snail shell crushing requires rather robust and sturdy LPJs (Meyer 1989, 1990a; Keenleyside 1991) and snails are substrate-associated, thus restricted to the benthic habitat (McCrary et al. 2008). LPJ morphology is closely associated with head and body shape in *A. tolteca* (Fig. 3, Fig. S2). Indeed, consistently throughout the species complex, benthic Midas cichlids have thicker and more robust horns than their limnetic counterparts (Meyer 1990a; Barluenga et al. 2006). These and other ecologically relevant traits should be responsive to disruptive selection, given their parallel evolution across lakes and demonstrated partial genetic basis (Lu and Bernatchez 1999; Peichel et al. 2001; Elmer et al. 2010a; Manousaki et al. 2013; Franchini et al. in press).

However, Midas cichlids also respond plastically to different environments so phenotypic plasticity likely also contributes to the documented eco-morphological variation in LPJs and body shape. In Midas cichlids and other freshwater fishes, LPJ morphology, and to a similar extent probably also body shape, respond plastically during ontogeny according to feeding mode (Meyer 1987, 1990a,b1990b; Muschick et al. 2011; Gunter et al. 2013). Even if the phenotypic signature of individual specialization we have identified is partially caused by plasticity, that does not contradict a specialization along the benthic-limnetic axis that can precede speciation. In fact plasticity likely plays a key role in the evolution from generalist to specialists (e.g. Adams and Huntingford 2004; Pfennig et al. 2010).

### A case of incipient sympatric diversification?

Our main finding, that morphology is closely linked to ecological resource use in this isolated Midas cichlid species, indicates that *A. tolteca* individuals are locally adapted and specialized in their habitat exploitation along the benthic-limnetic axis (Matthews et al. 2010). We have shown that a variable generalist species can also include a wide range of individual specialists. Specialization is a necessary ingredient for adaptive radiation; ecological speciation theory predicts a bimodal distribution of ecologically relevant traits when disruptive selection is at work toward sympatric speciation (Schluter 2000; Bolnick 2011; Nosil 2012). Yet despite the extent of observed variation, the eco-morphological traits we investigated were continuously distributed (Table 1). These results seem to reject the possibility of discrete eco-morphs in *A. tolteca* (Elmer et al. 2010a). Similarly, we did not find any neutral genetic sub-structuring in our sample of *A. tolteca* specimens that might indicate signatures of assortative mating by ecotype or other subpopulation structuring based on this set of microsatellite markers. Indeed, genetic diversity in Asososca Managua is low (0.549) compared to other crater lakes that are known to house multiple described species [Apoyo: 0.590, Xiloá: 0.668 (Barluenga and Meyer 2010)]. While we do not know whether the observed individual specialization is due to traits that are genetically fixed, phenotypically plastic, or a combination of both, in the face of strong divergent selection between habitats this phenotypic variation might eventually lead to divergence and result in reproductively isolated eco-morphs (Schluter 2000).

It has been hypothesized that adaptive radiation and speciation (in cichlids) proceed by the following stages: niche-use divergence into macrohabitats, followed by further ecomorphological divergence, and finally differentiation based on traits relevant for communication (Streelman and Danley 2003; Kocher 2004; Gavrilets and Losos 2009). More generally, speciation is often seen as a continuous process with several intermediate stages differing in the degree of adaptive ecological variation and reproductive isolation (Hendry et al. 2009a). In light of the reported parallel evolution along the benthic–limnetic axis in Midas cichlids, the individual specialization reported here might become essential for any future intralacustrine divergence and makes *A. tolteca* a candidate model system for investigating the evolutionary stages that precede lineage diversification.

## Supporting Information

Figure S1 Definition of eco-morphological measurements.

Figure S2 PCA of standardized eco-morphological variables.

Table S1 Summary data of microsatellite loci.
