# Supplementary figures and images for: Crater lake cichlids individually specialize along the benthic–limnetic axis
Source: Ecol Evol. 2014 Mar 7;4(7):1127–39. doi: 10.1002/ece3.1015 (PMC3997327; doi:10.1002/ece3.1015)

(A)

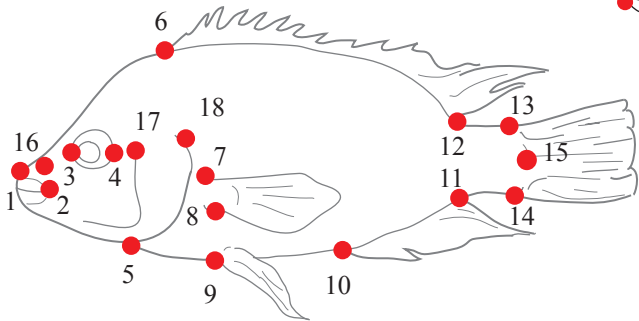

(B)

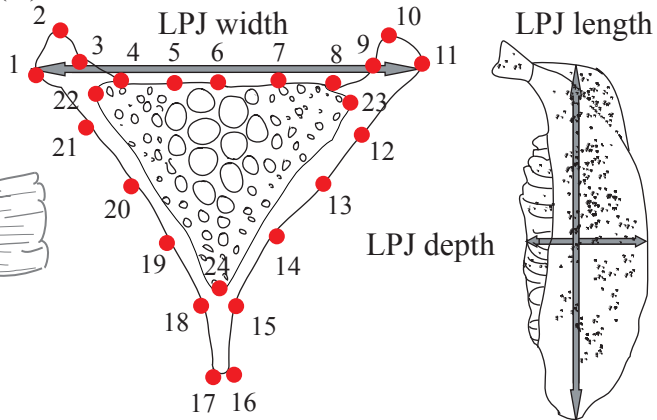

Supplement: Figure S1 — Definition of eco-morphological measurements. [file ece30004-1127-sd1.pdf]

(A)

|            | PC 1   | PC 2   |
|------------|--------|--------|
| BHI        | -0.272 | 0.437  |
| LPJ weight | -0.455 | -0.19  |
| LPJ width  | -0.446 | <-0.1  |
| LPJ length | -0.393 | -0.181 |
| LPJ depth  | -0.405 | <-0.1  |
| PC1Body    | -0.237 | 0.468  |
| PC2Body    | <0.1   | 0.499  |
| PC3Body    | -0.133 | 0.391  |
| d15N       | 0.2    | 0.334  |
| d13C       | -0.293 | <-0.1  |

(B)

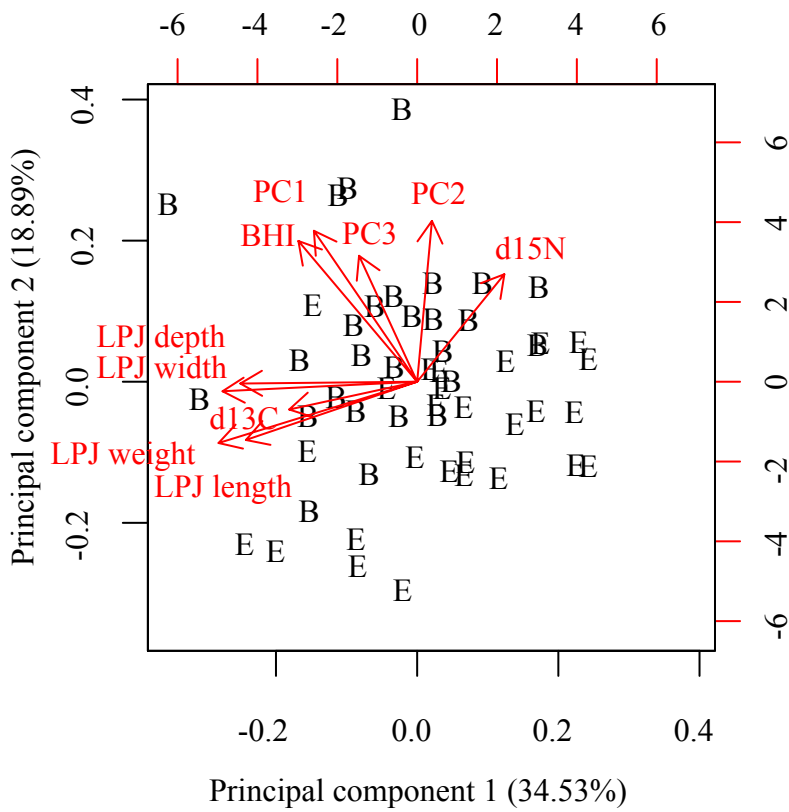

Supplement: Figure S2 — PCA of standardized eco-morphological variables. [file ece30004-1127-sd2.pdf]
