# Supplementary material for: Crater lake cichlids individually specialize along the benthic–limnetic axis
Source: Ecol Evol. 2014 Mar 7;4(7):1127–39. doi: 10.1002/ece3.1015 (PMC3997327; doi:10.1002/ece3.1015)
Supplement: Table S1 — Summary data of microsatellite loci. [file ece30004-1127-sd3.docx]

**Summary data per microsatellite locus.**

|  | **ABUR 151** | **ABUR 82** | **M1M** | **UNH011** | **UNH0012** | **M2** | **M7** | **TMOM7** | **ABUR45** | **BURTKIT** | **M12** | **UNH013** | **UNH002** |
| --- | --- | --- | --- | --- | --- | --- | --- | --- | --- | --- | --- | --- | --- |
| sample size (*n*) | 109 | 102 | 110 | 109 | 94 | 113 | 110 | 112 | 113 | 111 | 116 | 112 | 106 |
| number of alleles (*Na*) | 2 | 6 | 3 | 3 | 9 | 13 | 14 | 4 | 5 | 9 | 6 | 14 | 9 |
| number of effective alleles (*Ne*) | 1.037 | 2.094 | 1.622 | 1.990 | 2.220 | 5.163 | 9.286 | 1.671 | 1.907 | 2.528 | 2.214 | 4.523 | 2.554 |
| Observed Heterozygosity (*Ho*) | 0.018 | 0.451 | 0.336 | 0.550 | 0.596 | 0.894 | 0.882 | 0.420 | 0.407 | 0.586 | 0.552 | 0.821 | 0.566 |
| Gene diversity/Expected Heterozygosity (*He*) | 0.036 | 0.522 | 0.383 | 0.497 | 0.550 | 0.806 | 0.892 | 0.401 | 0.476 | 0.604 | 0.548 | 0.779 | 0.608 |
| Fixation Index (*Fis*) | 0.491 | 0.137 | 0.123 | -0.107 | -0.084 | -0.108 | 0.012 | -0.045 | 0.144 | 0.031 | -0.006 | -0.055 | 0.070 |
